# Sequence Homology at the Breakpoint and Clinical Phenotype of Mitochondrial DNA Deletion Syndromes

**DOI:** 10.1371/journal.pone.0015687

**Published:** 2010-12-20

**Authors:** Bekim Sadikovic, Jing Wang, Ayman El-Hattab, Megan Landsverk, Ganka Douglas, Ellen K. Brundage, William J. Craigen, Eric S. Schmitt, Lee-Jun C. Wong

**Affiliations:** Molecular and Human Genetics, Baylor College of Medicine, Houston, Texas, United States of America; King's College London, United Kingdom

## Abstract

Mitochondrial DNA (mtDNA) deletions are a common cause of mitochondrial disorders. Large mtDNA deletions can lead to a broad spectrum of clinical features with different age of onset, ranging from mild mitochondrial myopathies (MM), progressive external ophthalmoplegia (PEO), and Kearns-Sayre syndrome (KSS), to severe Pearson syndrome. The aim of this study is to investigate the molecular signatures surrounding the deletion breakpoints and their association with the clinical phenotype and age at onset. MtDNA deletions in 67 patients were characterized using array comparative genomic hybridization (aCGH) followed by PCR-sequencing of the deletion junctions. Sequence homology including both perfect and imperfect short repeats flanking the deletion regions were analyzed and correlated with clinical features and patients' age group. In all age groups, there was a significant increase in sequence homology flanking the deletion compared to mtDNA background. The youngest patient group (<6 years old) showed a diffused pattern of deletion distribution in size and locations, with a significantly lower sequence homology flanking the deletion, and the highest percentage of deletion mutant heteroplasmy. The older age groups showed rather discrete pattern of deletions with 44% of all patients over 6 years old carrying the most common 5 kb mtDNA deletion, which was found mostly in muscle specimens (22/41). Only 15% (3/20) of the young patients (<6 years old) carry the 5 kb common deletion, which is usually present in blood rather than muscle. This group of patients predominantly (16 out of 17) exhibit multisystem disorder and/or Pearson syndrome, while older patients had predominantly neuromuscular manifestations including KSS, PEO, and MM. In conclusion, sequence homology at the deletion flanking regions is a consistent feature of mtDNA deletions. Decreased levels of sequence homology and increased levels of deletion mutant heteroplasmy appear to correlate with earlier onset and more severe disease with multisystem involvement.

## Introduction

Mitochondria are energy producing cellular organelles that contain their own genetic material, mitochondrial DNA (mtDNA). Depending on energy demand of various tissues, there are hundreds to thousands of mitochondria per cell, and each mitochondrion contains 2–10 mtDNA molecules. Defects in mtDNA cause mitochondrial disorders, predominantly affecting tissues of high energy demand such as muscle and nerve. The disease severity, in general, depends on the degree of mutant heteroplasmy (mixture of wild type and mutant mtDNA molecules within a cell) in affected tissues. Mitochondrial DNA deletions are a common cause of mitochondrial disease and may contribute to the process of normal aging [1]. MtDNA deletions were first discovered in the skeletal muscle of patients with mitochondrial myopathies (MM) and Kearns-Sayre syndrome (KSS) [2], [3], [4]. These patients typically present with progressive external opthalmoplegia (PEO), pigmentary retinopathy, and one or more of the following: cerebellar ataxia, a cardiac conduction defect, and elevated protein concentration in the cerebrospinal fluid [5]. Although onset before the age of 20 is one of the diagnostic criteria, many patients with PEO or MM without other KSS symptoms often develop neuromuscular symptoms at a much older age [6]. In contrast, symptoms in young children and infants can be quite heterogeneous and severe. Many of these patients develop Pearson syndrome, which is characterized by infantile onset of sideroblastic anemia, with vacuolization of bone marrow precursor cells and pancreatic dysfunction [7]. Patients who survive infancy may develop KSS at a later age. Young patients often exhibit a multisystem clinical presentation involving the neuromuscular, hematologic, gastrointestinal, and metabolic systems as well as growth failure. In contrast, patients with adult onset mtDNA deletion syndromes exhibit mostly neuromuscular and ophthalmologic symptoms [8]. Diagnostically, in young patients it is possible to use the less invasive blood samples for mtDNA deletion testing since the mtDNA molecules carrying deletions are expected to be present in all tissues due to the severe multisystem disorder. The deletion in young patients is most likely a *de novo* germline or early embryonic event, whereas in older patients, the mtDNA deletion most likely occurs somatically in the affected tissue. Thus, the deletion mtDNA molecules are usually not present in blood specimens and it is necessary to use muscle for the detection of mtDNA deletion [8].

At the molecular level, the majority (approximately 60%) of mtDNA deletions occur at a region that is flanked by short direct repeat sequences, one of which is usually lost during the deletion process, and are referred to as class I deletions [9], [10]. Approximately 30% of mtDNA deletions have been shown to be flanked by imperfect repeats containing a few mismatches (class II deletions), and about 10% have no repeats at the deletion flanking regions [11]. The most common mtDNA deletion is a large 5 kb deletion (m.8470-m.13447), which is present in approximately one third of patients, and is flanked by a 13 nt class I direct repeat [10]. Such repeats are thought to play a role in the formation of mtDNA deletions. However, it is unclear what biological mechanisms are directly responsible for these events. It is speculated that defects in mtDNA replication caused by inappropriate alignment of direct repeats may be the cause of mtDNA deletions [12], [13], [14]. An alternative method involving the repair of mtDNA damage has recently been proposed [15].

While the presence of an mtDNA deletion is a common feature in patients with mtDNA deletion syndrome, the clinical features can range from very mild, late-onset disorders affecting predominantly neuromuscular tissues to very severe, multisystem disease in infants who often succumb to the disease [8]. The objective of this study was to characterize the molecular features associated with mtDNA deletion syndromes, including levels of heteroplasmy, locations of deletions, presence of direct repeats, degree of sequence homology at the breakpoints, and to correlate them with the clinical features and age at diagnosis in a cohort of 67 patients with mtDNA deletion syndrome.

## Materials and Methods

### Patient samples

This study involved the review and analysis of the clinical information and molecular results of 67 patients who were diagnosed with a mtDNA deletion syndrome. The detailed clinical information and diagnoses are tabulated for each patient (Table S1). The study was performed according to an Institutional Review Board protocol for research on human subjects at the Baylor College of Medicine.

### Analysis of mtDNA deletions

Extracted DNA samples from blood or muscle were analyzed by Southern blot for mtDNA deletions [16], [17]. A majority of the DNA samples were also analyzed by oligonucleotide array comparative hybridization (aCGH) with the MitoMet® platform, as previously described [18], in order to determine the location and size of the deletion, as well as the degree of mtDNA deletion heteroplasmy. In a small number of the cases, the level of heteroplasmy was determined by densitometric analysis of the Southern blot, the results of which were shown to be consistent with the quantification by the MitoMet® aCGH method [18]. The deletion junction sequences of all samples were further analyzed by PCR amplification across the deletion junction, followed by sequence analysis using the BigDye Terminator Cycle Sequencing kit (version 3.1) and the ABI3730XL automated DNA sequencer with Mutation Surveyor V3.20 software (Softgenetics, State College, CA). A representative sample is shown in Figure S1. The detailed 5′ and 3′ sequence position flanking the deletion region and heteroplasmy data are tabulated for each sample (Table S1.).

### Analysis of sequence homology around the deletion junction

Sequences of a total of 49 nucleotides surrounding the deletion junction were aligned to proximal and distal sequences, 5′ and 3′ to the deletion region, respectively. Since the majority of deletion junctions contain a short direct repeat that appears at both 5′ and 3′ sides of the junction, it is not possible to determine the exact breakpoint. We therefore arbitrarily assign the breakpoint nucleotide to the most 5′ nucleotide of the short repeat. The breakpoint nucleotide is easier to assign in cases where there is zero or single nucleotide overlap. The sequence homology in the 49 nt region (24 nt on each side of the breakpoint nucleotide) of the proximal and distal sequences was analyzed using ClustalW software [19] with default parameters (http://www.ebi.ac.uk/Tools/clustalw2/index.html). The choice of 24 nt on each side of the breakpoint nucleotide is based on a recent review by McVey and Lee, which shows that microhomology mediated end joining (MMEJ) repair, a deletion-causing mechanism involving largest regions of imperfect microhomology, includes perfect and imperfect repeats ranging from 5 to 25 nucleotides flanking the breakpoint [20]. The presence of a direct repeat at the breakpoint, and the overall homology (represented as percent of total number of homologous nucleotides in the alignment), were scored and annotated (Table S1). In addition to the breakpoint sequence analysis, analysis of pair-wise alignments of 49 nt in 12 regions spanning the area of the majority of mtDNA deletions at thousand nucleotide increments, starting at m.4500 position and ending at m.15500, was also performed. We included the full range of possible iterations for a total of 66 alignments.

### Statistical Analysis

We used a homoscedastic Student's t-Test with 2-tailed distribution to determine significance in the comparison of levels of heteroplasmy in relation to age groups and the analysis of breakpoint homology in patients relative to random alignments. The comparison of categorical data (presence or absence of the common deletion) in blood versus muscle tissue was analyzed using Fisher's exact test. Differences were considered significant at P<0.05.

## Results

Sixty seven patients found to have an mtDNA deletion were grouped according to age at diagnosis into 4 age groups; 0–5, 6–20, 21–40, and older than 41 years-old for groups 1 to 4 respectively. There were 17, 18, 19, and 13 patients in each age group, respectively. Clinical features of the patients were reviewed, and are presented in detail in Table S1. A custom designed, clinically validated MitoMet® array CGH was used to estimate the deletion breakpoints and levels of heteroplasmy, followed by PCR/DNA sequencing to identify the exact breakpoints and deletion sizes (Figure S1 and Table S1). In some of the samples that were analyzed prior to the availability of the MitoMet® array, the percent heteroplasmy was estimated by densitometric analysis of the Southern blots.

**Figure 1:**
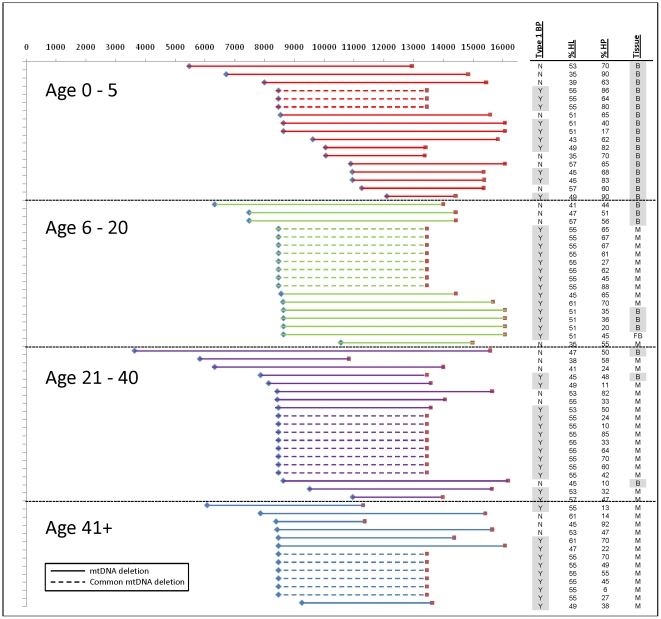
Distribution of deletions in age-grouped mtDNA deletion patients. X-axis represents the nucleotide position along the mitochondrial genome, and y-axis shows deletions for individual patients arranged by the age-group and the most proximity of the left breakpoint to the 1^st^ nucleotide, from top to bottom. Blue diamonds and red squares represent the left and right breakpoints respectively. Each patient sample has, in the columns on the right, description of the presence of type 1 breakpoint (Type I BP), percent heteroplasmy (% HP), percentage of sequence homology at breakpoints (% HL), and tissue type (Tissue).

**Figure 2:**
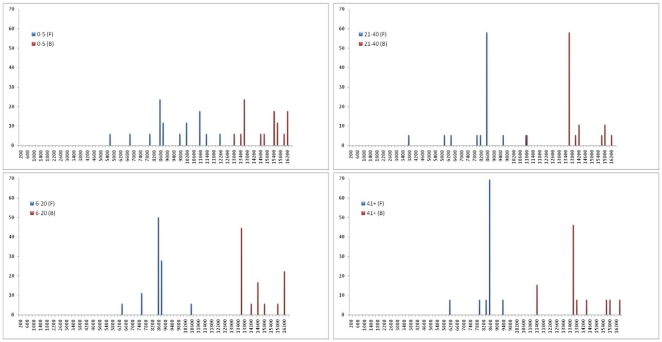
Breakpoint distribution in age-grouped mtDNA deletion patients. X-axis represents the nucleotide position along the mitochondrial genome in 200 nucleotide increments, and y-axis represents the percentage of samples with that breakpoint in BP1 and BP2 to the total number of breakpoints in BP1 and BP2 respectively (BP1 and BP2 refer to the breakpoint locations rounded to the next 200 nt increment).

To investigate the locations of the deletion breakpoints among the four age groups, the 5′ and 3′ deletion breakpoints of mtDNA deletion patients in each age group were mapped to the mitochondrial genome (Figure 1). The results showed an uneven distribution. Groups 2, 3, and 4 had a very similar pattern of deletion distribution, with the majority located near or at the common deletion breakpoint (8470–13447 nt). The youngest age group showed deviation from this common trend (Figure 1). As shown in Figure 2, the distribution of breakpoints in age groups 2, 3, and 4 has a few discrete locations with the most common breakpoint region accounting for 50–70% of overall breakpoints. Whereas group 1 patients showed a high heterogeneity of breakpoints and a diffused distribution, with the common breakpoint region accounting for no more than 25% of overall breakpoints. However, due to the small number of samples analyzed, the observed difference in breakpoint distribution between group 1 and other groups was not statistically significant. The size of deletion and the number of genes deleted were similar in all age groups (Table 1). In contrast, levels of deletion mutant heteroplasmy showed a decreasing trend with increasing age, with group 1 having an average of 67.9% heteroplasmy, which is significantly higher than that of the other groups combined (42–53% average, P = 0.00069). Group 1 patient specimens were all blood tissue, whereas the rest of the groups specimens were predominantly (82%) muscle tissue.

**Table 1:** Analysis of molecular features of mtDNA deletions in patients separated according to age groups.

Group	Age	Heteroplasmy (%)	Deletion size range and average (Kb)	Deleted genes (#)	Type I breakpoints	Homology at breakpoint	Samples with common 5 kb deletion
1	0 – 5	67.9	5.47–16.07 (5.48)	12.5	58.8 (10/17)	48.5	17.6
2	6 – 20	53.3	6.33–16.07 (6.02)	13.7	77.8 (14/18)	51.7	44.4
3	21 – 40	43.8	3.64–16.17 (5.79)	13.6	68.4 (13/19)	51.4	42.1
4	41+	42.2	6.07–16.07 (5.44)	12.7	76.9 (10/13)	53.9	46.1

**Figure 3:**
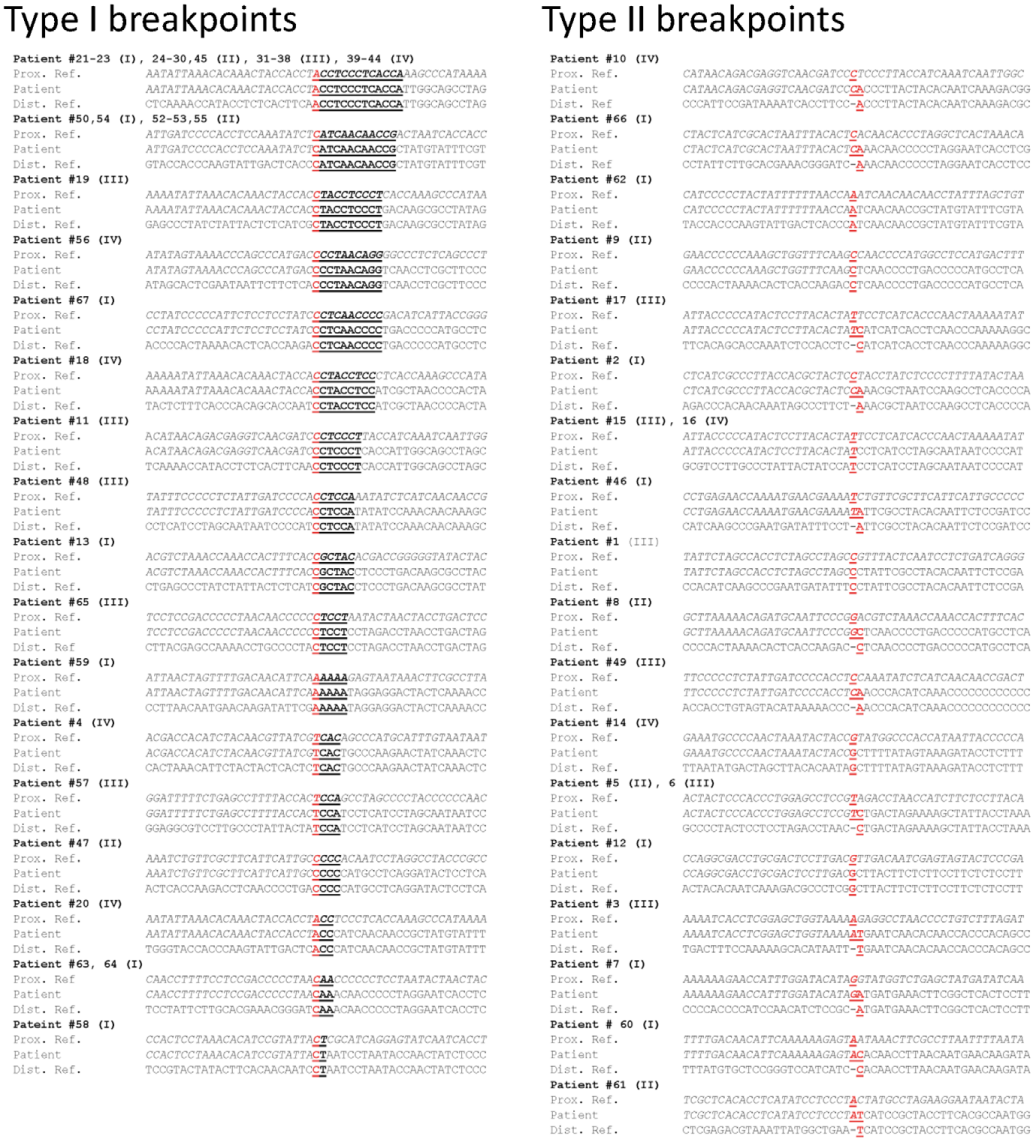
Sequence alignments of mtDNA deletion breakpoints. The two breakpoints in each mtDNA deletion sample (Prox. and Dist. Ref.) were aligned using ClustalW alignment tool. Alignments were grouped based on the location of the breakpoint as either within a direct repeat sequence (type I), or not within a direct repeat sequence (type II). Direct repeats are bolded and underlined. Mapped breakpoint nucleotides are in red. Top strand in each alignment is the proximal breakpoint sequence and bottom is the distal breakpoint sequence from Table S1. Alignments corresponding to the individual patient case from Table S1 are indicated to the left of the alignment.

**Figure 4:**
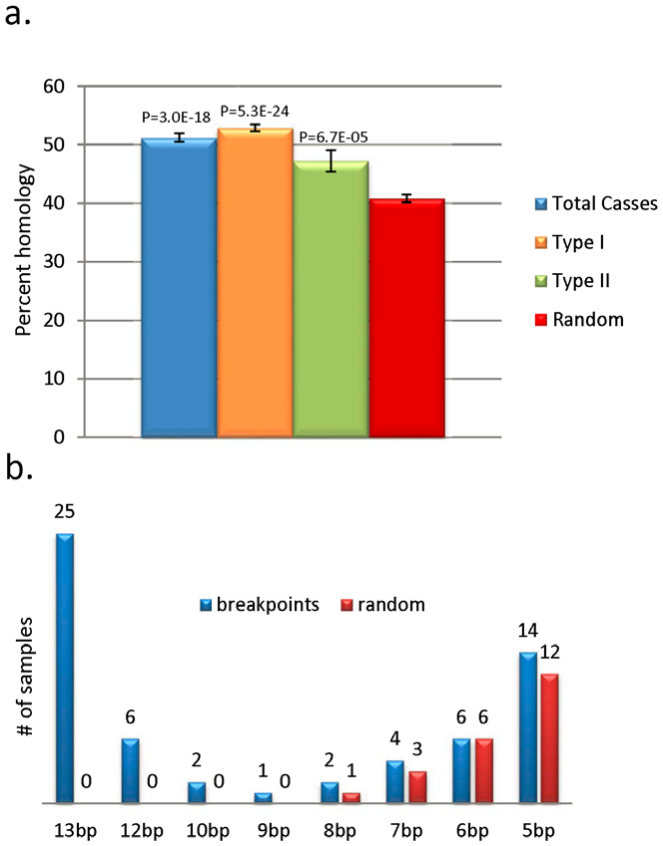
Analysis of homology at mtDNA deletion breakpoints relative to random alignment controls. A) Percent homology (percent of nucleotides in alignment out of total possible 49) inferred by ClustalW at mtDNA deletion breakpoints relative to random alignment background. The homoscedastic Student's t-Test with 2-tailed distribution was used to determine significance. B) Quantification of direct repeats at breakpoints (+/− 25 nt) in mtDNA deletion breakpoints relative to random alignment background.

**Figure 5:**
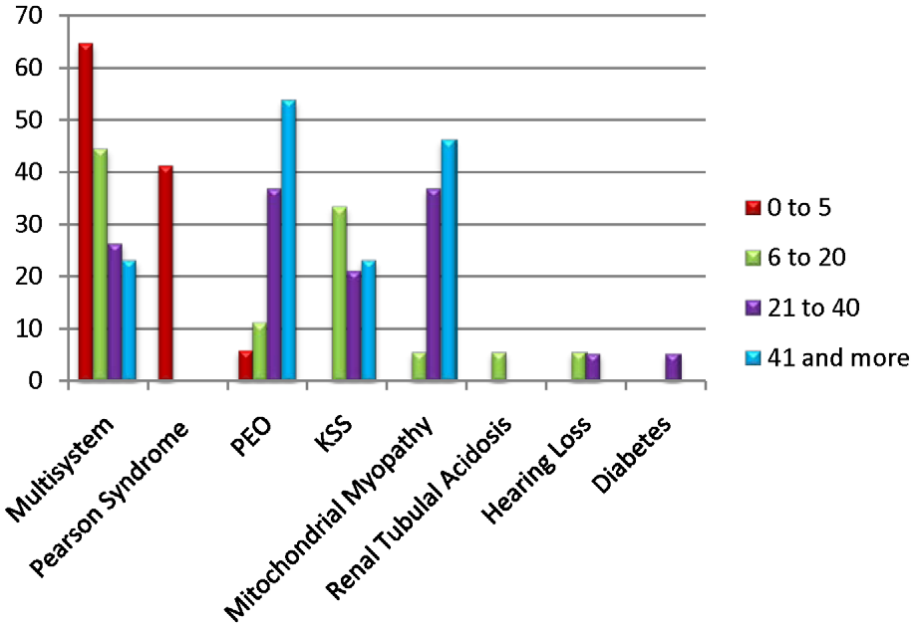
Clinical diagnosis and clinical manifestations in patients with mtDNA deletions. Y axix indicates the percentages of samples with a specific clinical diagnosis indicated in Table S1.

The molecular characteristics of DNA sequences at the deletion breakpoints were investigated by alignment (Figure 3) and analysis of DNA sequences (49 nt total including 24 nt on each side of the breakpoint plus the breakpoint nucleotide) surrounding the two breakpoints of each sample using ClustalW software (Table S1, Figure S2). This analysis shows the presence of two types of breakpoints: those involving and not involving a direct repeat. Seventy percent of mtDNA deletions involved a direct repeat sequence (Type I). The “common deletion” involving the 13 nt direct repeat at the breakpoint was present in 25 patients (25/67 = 37%). The second longest direct repeat (12 nt) involving the second most common deletion was observed in 6 patients. Half of the patients who had no direct repeat at the breakpoint had large direct repeats (more than 5 nt) near the breakpoint. ClustalW alignment tool was used to estimate the sequence homology, defined as the percentage of homologous nucleotides, in the 49 nucleotides alignment of the proximal and distal sequences surrounding the breakpoints of each mtDNA deletion case (Table S1). Furthermore, a majority of samples, with or without direct repeat at the breakpoint, showed very high sequence homology, exceeding 60% in some cases, within the 49 nucleotides around the breakpoint (Table 1). In fact, ¾ of the mtDNA deletions had at least one 5 nt+ direct repeat surrounding the breakpoints. When compared to 66 randomly aligned 49 nucleotide regions throughout the mitochondrial genome the degree of sequence homology within the breakpoint region was significantly higher (Figure 4a). In addition, the patient cohort showed a striking enrichment for the presence of large (>8 nt) direct repeats at the breakpoints relative to the randomly aligned sequences (Figure 4b). This analysis revealed a significant increase (p = 3×10^−14^) in sequence homology around the mtDNA deletion breakpoints above that of the mitochondrial genomic sequence background. When analyzed independently, both Type I and Type II breakpoints showed significant increase in homology relative to random alignments. Type I breakpoints showed significant (p<5.3×10^−24^) increase in homology relative to random breakpoints (52.9% vs. 40.8%). Although not as high as Type I breakpoints, homology at Type II breakpoints (47.2%) was also significantly (p<6.7×10^−5^) increased compared to random alignments. When all age groups are compared, group 1 showed significantly (p = 0.04) lower levels of sequence homology compared to the other 3 groups combined (48.5% compared to 52.2%), and the smallest percentage of samples with direct repeats at breakpoints (58.8% compared to 74%) (Table 1). Furthermore, the common deletion was present in 18% of group 1 patients, and in 44, 42, and 46% of patients in groups 2, 3, and 4, respectively (Table 1). Due to the small group sizes, statistical analysis lacks power to determine if these differences are significant (p = 0.08). However, there was a significant difference (p = 0.0014) in the frequency of the common deletion in patients with multisystem disorders (where the deletion is detected in blood) compared to patients with neuromuscular/PEO/MM disorders (3/26 vs. 22/41 patients, or 11.5% vs. 53.6%) (Table S1). Clinical features were analyzed across all age groups. Consistently, the majority of young patients (group 1) presented with multisystem disorders or Pearson syndrome; whereas most (68 to 78%) of older patients (groups 2, 3, and 4) were diagnosed with MM, PEO, KSS, or multisystem involvement (Figure 5).

## Discussion

Clinical manifestations of mtDNA deletion syndromes range from acute, multisystem, and often lethal disorders in infants and young children, to relatively mild neuromuscular symptoms that are often of adult onset [8]. The underlying molecular features of these deletions are thought to play a role in the deletion process, and may also be related to the severity of the disorder. The aim of this study was to investigate the molecular characteristics of mtDNA deletions and their correlation to the age at onset and disease clinical phenotype, with the hypothesis that specific molecular signatures are associated with mtDNA deletion mechanism and thus clinical manifestation. We maintain a large clinical and molecular database of patients with mitochondrial disorders, including a large cohort of patients with an mtDNA deletion syndrome, which provided us with a unique opportunity to test this hypothesis.

It has been shown previously that mtDNA deletions exhibit enrichment for direct repeats at the breakpoints [2], [3], [5], [11], [13], [21], [22]. These data have been corroborated by many groups and resulted in stratifying these deletions as type I (with a direct repeat) or type II (imperfect or no direct repeat) in relation to sequences at the two breakpoints. While the presence of the repeat may play a role in molecular events leading to an mtDNA deletion, the precise mechanism is not clear. On the other hand, there are numerous reports of young children severely affected with multisystem mtDNA deletion syndrome who did not have direct repeats at the breakpoints [8]. This prompted us to characterize deletion breakpoints molecularly. Our data reveal that, regardless of the presence of a direct repeat, most mtDNA deletion regions have a significant increase in sequence homology surrounding the breakpoints. About one third of the patients in this study that had type II breakpoints nevertheless showed a significant increase in sequence homology relative to what would be expected by chance. These data provide evidence that sequence homology is the primary determinant of breakpoint distribution in mtDNA deletion syndromes. Since large direct repeats have the longest continuous stretches of sequence homology, they would be expected to be the most frequent sites of mtDNA deletion breakpoints. The most common mtDNA deletion, which is flanked by the longest (13 nt) direct repeat, is observed in more than one third (25/67 = 37%) of our patients. The second most common deletion is flanked by the 12 nt direct repeat in six of our patient samples. The presence of sequence homology at the breakpoints suggests its role not only in the generation of the break but also in the repair of mtDNA damage. It has been suggested that repeats drive breakpoint generation when there is an error in mtDNA replication due to inappropriate alignment of direct repeats [12], [13], [14], or a mtDNA damage [15]. Both, defects in mtDNA replication caused by inappropriate alignment of direct repeats [12], [13], [14] and mis-annealing of a single strand mtDNA molecule following double stranded breaks [15] require the presence of direct repeats or sequence homology.

Although significantly higher than random mitochondrial genome homology, the youngest age group 1 showed significantly lower breakpoint homology relative to the older age groups. Furthermore, group 1 patients harbored a significantly lower percentage of samples with type I breakpoints (Table 1), and almost 3-fold decreased incidence of the common 5 kb mtDNA deletion relative to other three age groups, as well as increased heterogeneity in breakpoint distribution. However, disease severity is not affected by the size of deletion and genes deleted. These data suggest that molecular events responsible for mtDNA deletions in young patients may differ from those found in older age groups. One possibility is that mtDNA deletions in these patients are inherited from maternal germ line mutations, or are acquired during early embryogenesis, while the mtDNA deletions in older age groups represent later somatic random events. This is consistent with the clinical data obtained from these patients, where the vast majority of young patients present with the multisystem disease, while older age groups predominantly display a KSS spectrum or myopathy phenotype. Significantly increased levels of heteroplasmy in young patients relative to the older age groups provide further evidence for this hypothesis. Consistent with an earlier embryonic occurrence of the deletions, we have previously shown that levels of heteroplasmy for mtDNA deletions in young patients are present at similar levels in blood, muscle, skin and other tissues [8], whereas mtDNA deletions are almost exclusively localized to neuromuscular tissues in older patients. Whether there is interplay between the tissue specificity and the molecular mechanism of mtDNA rearrangement is not clear. It is possible that proteins involved in DNA rearrangement are differentially expressed in rapidly dividing/actively differentiating cells and non-dividing muscle and brain cells.

There is a significant difference (p<0.0014) in the frequency of the common deletion in patients with multisystem disorders (where the deletion is detected in blood) compared to patients with neuromuscular/PEO/MM disorders (11.5 versus 53.6%), further suggesting a different molecular mechanism for mtDNA rearrangement between germ cells and somatic cells. MtDNA deletions in germ cells may be driven by DNA replication, while mtDNA deletions in postzygotic somatic cells may be due to mis-pairing of homologous regions during repair of random oxidative mtDNA damage.

A recent study of mtDNA deletions in aged human skeletal muscle fibers has reported an increased incidence of mtDNA deletions with the presence of direct repeats compared to no direct repeats at breakpoints in older (>60 years old) relative to younger individuals [23]. The authors proposed that oxidative-damage induced DNA-replication errors resulting in mtDNA deletions accumulated up to a pathological threshold over time. Presumably there would be a higher frequency of deletions at the direct-repeat hotspots that would selectively accumulate and be predominant over time. Conversely, a study of mtDNA deletion in substantia nigra neurons of age-matched patients with Parkinson Disease, mtDNA multiple deletion disorder, and single deletions, showed no difference in the type of deletion breakpoints in these samples, suggesting that a similar mechanism may be involved [24]. Krishnan and colleagues suggested that increased reactive oxygen species produced in these neurons could cause double strand breaks through DNA damage or replication stalling, and proposed aberrant DNA repair as a mechanism for mtDNA deletion formation [15]. Further support for this mechanism comes from a study in which restriction endonuclease *PstI*-induced breakpoints resulted in the formation of mtDNA deletions flanked by short sequences with or without direct repeats [25]. A common theme across all these studies is the detection of increased degree of sequence homology around the breakpoints of mtDNA deletions with breakpoints containing direct repeats, imperfect repeats, or no repeats at the breakpoint. Our data support these findings, and further demonstrate that a common feature of the breakpoints, irrespective of the type, is a significantly increased sequence homology around the breakpoint. Similar to the DNA replication model of mtDNA deletions, DNA break repair models rely on miss-annealing of the short homologous sequences in the single stranded DNA molecule, which in the latter case would be generated via 3′ to 5′ exonuclease activity. Three major types of error-prone double-strand breakpoint repair in the context of genomic DNA include non-homologous endjoining (NHEJ), microhomology-mediated end joining (MMEJ), and single-strand annealing (SSA). NHEJ was shown to rely on small (1–4 nt) homologies resulting in small <5 nt deletions/insertions, while MMEJ involves larger (5–25) nucleotide homologies that may or may not be direct repeats and can result in larger deletions [20], [26], [27]. SSA requires direct repeats >30 nt. Therefore, MMEJ-type double-strand break repair mechanism would most closely fit the criteria based on the type of deletions seen in the mtDNA. The mitochondrial proteins that may be involved include mitochondrial polymerase gamma (POLG1) and SFN which are known to have a 3′ to 5′ exonuclease activity and are targeted to mitochondria [28], [29], [30]. Another mechanism named microhomology-mediated break-induced replication (MMBIR), involving small (2–5 nt) homologous sequences at or near the breakpoint junctions, was recently proposed to be involved in human copy number variance (CNV) formation, and could theoretically be involved in mtDNA deletions [31]. This mechanism is based on the assumption that during DNA replication, single stranded DNA breaks would generate single-stranded 3′ tails which could anneal with any single-stranded homologous DNA nearby and thereby would induce DNA rearrangements.

In conclusion, breakpoint sequence homology is a consistent feature of most mtDNA deletions. These data also suggest that the underlying molecular signatures determine the mtDNA deletion mechanism and may also correlate with clinical manifestations in patients with a mtDNA deletion syndrome. While we are still far from fully understanding the molecular mechanisms underlying these disorders, certain specific molecular features such as increased percentage of heteroplasmy, relatively lower homology at breakpoints, and a decreased frequency of the common deletions, may play a role in the severity and earlier age of onset of mtDNA deletion syndromes.

## Supporting Information

Figure S1 **Mapping of mtDNA breakpoints.** A) The position of the mtDNA deletion and level of heteroplasmy was determined using the clinical MitoMet array using a previously published protocol [18]. Y-axis shows the position of the probes along the mitochondrial chromosome. The location and the level of heteroplasmy was calculated based on normalized probe intensity to age and tissue matched controls. B) Based on the MitoMet results mtDNA was PCR amplified and sequenced across the breakpoint to determine the precise nucleotides involved in the breakpoint. Top sequence represents sequence 5′ to the breakpoint and bottom sequence represents the sequence 3′ to the breakpoint. Middle sequence is sequence across the patient's breakpoint
(TIFF)

Figure S2 **Analysis of sequence homology at mtDNA deletion breakpoints.** The two breakpoints in each mtDNA deletion sample (Prox. and Dist. Ref.) were aligned using ClustalW alignment tool. Alignments were grouped based on the location of the breakpoint as either within a direct repeat sequence (type I), or not within a direct repeat sequence (type II). Direct repeats of 5+ nucleotides are highlighted (dark grey), and repeats of smaller size are highlighted (light gray). Mapped breakpoint nucleotides are in red. ClustalW alignments where the breakpoints do not align (in Type II breakpoints) are because, ClustalW alignment shifts the alignment to achieve maximum homology in that region. Top strand in each alignment is the proximal breakpoint sequence and bottom is the distal breakpoint sequence from Table S1. Alignments corresponding to the individual patient case from Table S1 are indicated to the left of the alignment.
(TIFF)

Table S1 The “breakpoint nucleotide” refers to the nucleotide position at which the breakpoint has occurred, either in the 5′ or the 3′ position. A majority of the subjects analyzed have either a single nucleotide, or a stretch of identical nucleotides (direct repeats), present at the two breakpoints flanking the deletion. As such, the sequence alignment of the breakpoints does not allow us to predict if the breakpoint happens at the 5′ or the 3′ of the overlapping nucleotide/s at the breakpoint 1 and 2 respectively. We refer to the common region of overlap as the “breakpoint nucleotide”. The breakpoint nucleotide is indicated in columns proximal breakpoint reference sequence (5′ to 3′) and distal breakpoint sequence (5′ to 3′) with “[]”. In cases with direct repeats at breakpoints, which are longer than 1 nucleotide, the most 5′ nucleotide of the direct repeat is indicated with the bracket. These correspond to the nucleotides that are labeled in red color in Figure 3 and Figure S2. The sequences in the Proximal breakpoint reference sequence column which are 3′ from the breakpoint nucleotide, and sequences in the Distal breakpoint sequence column which are 5′ from the breakpoint nucleotide represent the 5′ and 3′ sequences of the deleted molecule respectively. In cases where there is not a common nucleotide(s) at the two breakpoints, the sequence breakpoint is predicted to be 3′ to the breakpoint nucleotide in the breakpoint 1 sequence, and 5′ to the breakpoint nucleotide in the breakpoint 2 sequence. All sequences are presented in 5′ to 3′ direction, left to right.
(XLSX)
