# Correction: Sequence Homology at the Breakpoint and Clinical Phenotype of Mitochondrial DNA Deletion Syndromes

**DOI:** 10.1371/journal.pone.0188610

**Published:** 2017-11-20

**Authors:** Bekim Sadikovic, Jing Wang, Ayman W. El-Hattab, Megan Landsverk, Ganka Douglas, Ellen K. Brundage, William J. Craigen, Eric S. Schmitt, Lee-Jun C. Wong

The middle initial of the third author is missing. The third author’s complete name is: Ayman W. El-Hattab. The correct citation is: Sadikovic B, Wang J, El-Hattab AW, Landsverk M, Douglas G, Brundage EK, et al. (2010) Sequence Homology at the Breakpoint and Clinical Phenotype of Mitochondrial DNA Deletion Syndromes. PLoS ONE 5(12): e15687. https://doi.org/10.1371/journal.pone.0015687
