# Supplementary figures and images for: Sequence Homology at the Breakpoint and Clinical Phenotype of Mitochondrial DNA Deletion Syndromes
Source: PLoS One. 2010 Dec 20;5(12):e15687. doi: 10.1371/journal.pone.0015687 (PMC3004954; doi:10.1371/journal.pone.0015687)

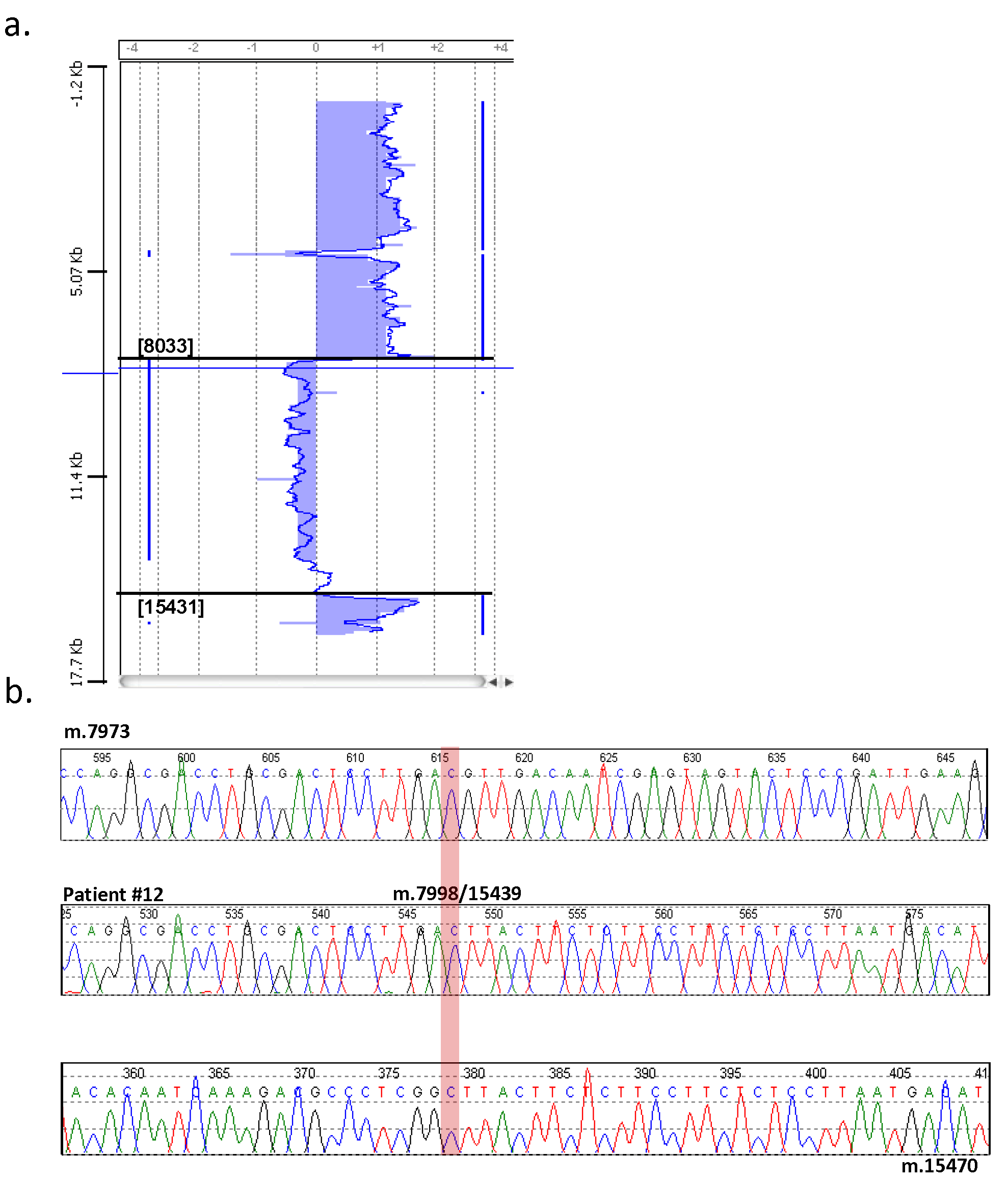

Supplement: Figure S1 — Mapping of mtDNA breakpoints. A) The position of the mtDNA deletion and level of heteroplasmy was determined using the clinical MitoMet array using a previously published protocol [18]. Y-axis shows the position of the probes along the mitochondrial chromosome. The location and the level of heteroplasmy was calculated based on normalized probe intensity to age and tissue matched controls. B) Based on the MitoMet results mtDNA was PCR amplified and sequenced across the breakpoint to determine the precise nucleotides involved in the breakpoint. Top sequence represents sequence 5′ to the breakpoint and bottom sequence represents the sequence 3′ to the breakpoint. Middle sequence is sequence across the patient's breakpoint (TIFF) [file pone.0015687.s001.tiff]

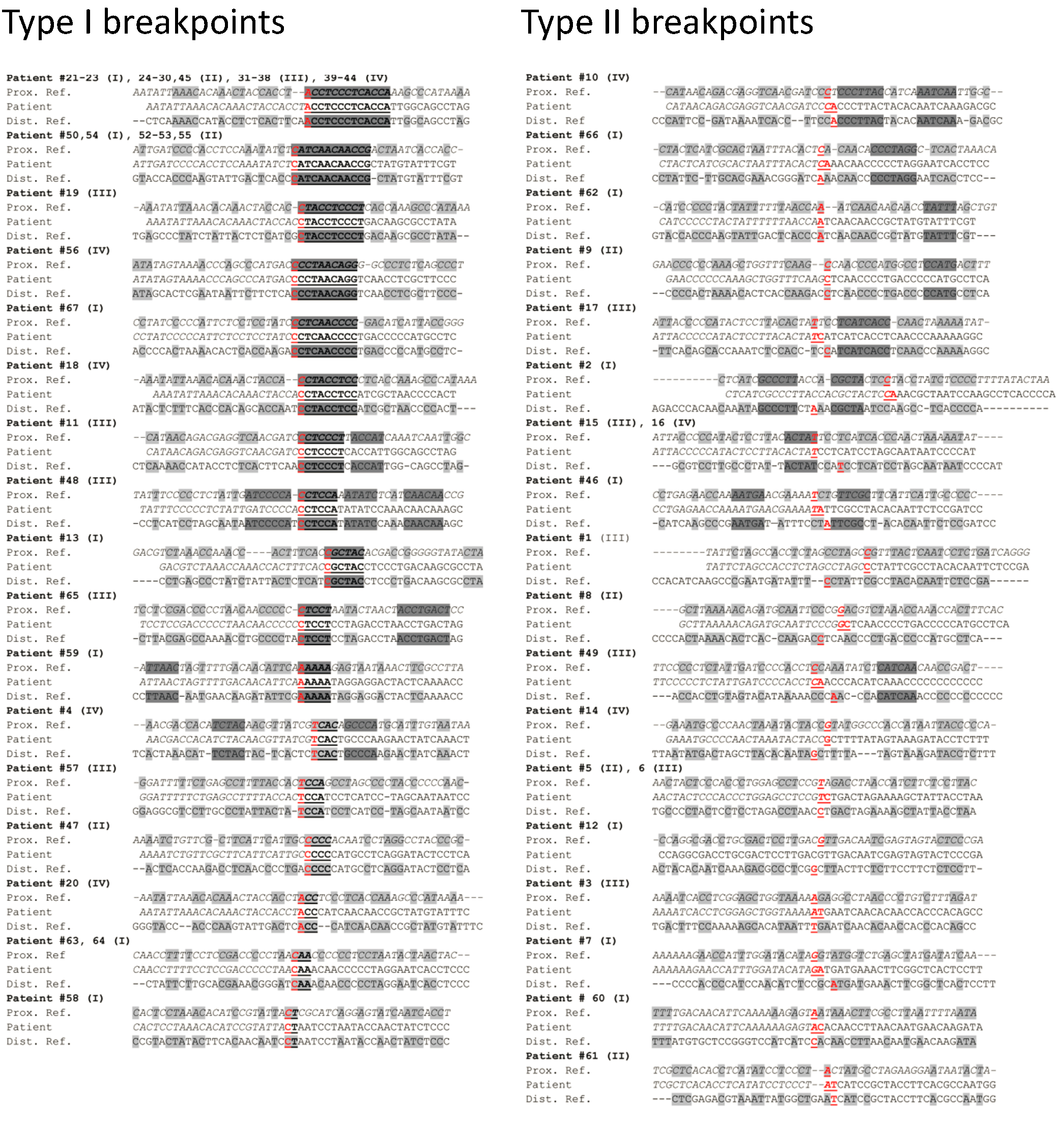

Supplement: Figure S2 — Analysis of sequence homology at mtDNA deletion breakpoints. The two breakpoints in each mtDNA deletion sample (Prox. and Dist. Ref.) were aligned using ClustalW alignment tool. Alignments were grouped based on the location of the breakpoint as either within a direct repeat sequence (type I), or not within a direct repeat sequence (type II). Direct repeats of 5+ nucleotides are highlighted (dark grey), and repeats of smaller size are highlighted (light gray). Mapped breakpoint nucleotides are in red. ClustalW alignments where the breakpoints do not align (in Type II breakpoints) are because, ClustalW alignment shifts the alignment to achieve maximum homology in that region. Top strand in each alignment is the proximal breakpoint sequence and bottom is the distal breakpoint sequence from Table S1. Alignments corresponding to the individual patient case from Table S1 are indicated to the left of the alignment. (TIFF) [file pone.0015687.s002.tiff]
